# Preoperative Risk Factors for Dry Eye Symptoms After Cataract Surgery: Femtosecond Laser-Assisted Cataract Surgery (FLACS) Versus Standard Cataract Surgery (SCS)

**DOI:** 10.3390/jcm14197091

**Published:** 2025-10-08

**Authors:** Elvia Mastrogiuseppe, Luca Lucchino, Francesca Giovannetti, Mattia D’Andrea, Davide Mastromarino, Alice Bruscolini, Alessandro Lambiase, Marco Marenco

**Affiliations:** Department of Sense Organs, Sapienza University of Rome, Viale del Policlinico 155, 00161 Rome, Italy; elvia.mastrogiuseppe@uniroma1.it (E.M.); luca.lucchino@uniroma1.it (L.L.); mattia.dandrea@uniroma1.it (M.D.); davide.mastromarino@uniroma1.it (D.M.); alice.bruscolini@uniroma1.it (A.B.); alessandro.lambiase@uniroma1.it (A.L.); marco.marenco@uniroma1.it (M.M.)

**Keywords:** Femtosecond Laser-Assisted Cataract Surgery (FLACS), Standard cataract surgery (SCS), Dry eye disease (DED), Ocular Surface Disease Index (OSDI), Schirmer Test I, Oxford score

## Abstract

**Background:** Despite technological advancements in cataract surgery, including Femtosecond Laser-Assisted Cataract Surgery (FLACS), postoperative dry eye disease (DED) remains a challenge, impacting patients’ quality of life. Identifying preoperative predictors of ocular discomfort could improve patient management. **Methods:** This exploratory prospective study compared the onset of DED symptoms and ocular surface changes after FLACS and standard cataract surgery (SCS). Twenty eyes were evaluated preoperatively and postoperatively, using Ocular Surface Disease Index (OSDI), Non-Invasive Break-Up Time (NI-BUT), Schirmer I Test, and Oxford Score. One-week OSDI was analyzed as the dependent variable using multivariable quantile regression (τ = 0.5), with baseline parameters (OSDI, Oxford score, Schirmer test, NI-BUT), age, BCVA, surgical technique, and cumulative dissipated energy (CDE) as predictors. **Results:** FLACS was associated with a transient worsening of OSDI at one week, which resolved by three months, whereas SCS showed a milder but more gradual increase. In multivariable analysis, baseline OSDI (β = 0.61, *p* < 0.001) and Oxford score (β = 5.42, *p* = 0.045) were independent predictors, while surgical technique and perioperative parameters were not significant. In a reduced model, both predictors confirmed their association. Subgroup analyses showed baseline OSDI as predictive only in FLACS. **Conclusions:** Preoperative ocular surface status emerges as the main determinant of early postoperative DED symptoms. Routine assessment of OSDI and Oxford scores may help identify at-risk patients and guide preventive strategies.

## 1. Introduction

Cataract surgery stands as the most performed procedure worldwide [1,2]. Thanks to advancements in modern technology, this surgery has become increasingly safe with very few complications related to the execution of the procedure [3,4]. Consequently, the most prevalent post-operative concern is ocular surface discomfort, due to dry eye disease (DED) [5,6]. A recent meta-analysis [7] reveals that, following cataract surgery, DED affects 37.4% of patients without a prior history of dry eye related symptoms. Despite increasing surgical expertise, DED can significantly impact patients’ quality of life postoperatively [8]. In addition, ocular surface alterations following cataract surgery can induce or exacerbate pre-existing DED, impairing visual outcomes [9,10].

Since its introduction in 2010, femtosecond laser-assisted cataract surgery (FLACS) has improved accuracy and efficiency in cataract treatment. FLACS allows for precise corneal incisions, highly accurate anterior capsulotomy, and controlled lens fragmentation. As previously demonstrated [11,12], this approach significantly reduces ultrasound (US) energy consumption and shortens phacoemulsification time, potentially lowering postoperative inflammation and decreasing the risk of DED. However, the direct interaction between the ocular surface and the vacuum system, combined with the sustained pressure from the suction ring, may cause microscopic damage to the sensitive ocular surface. Furthermore, the laser procedures used in FLACS may negatively impact tear film stability [9,13]. These factors can ultimately contribute to an increased risk of DED [14]. Recent evidence further supports this concern: a review reported that DED is highly prevalent after FLACS, with symptoms peaking at one week and improving by three months, mainly due to suction ring and patient interface-related stress [15].

DED severity typically peaks in the first postoperative week and may persist from one month to over a year [7]. Moreover, a recent meta-analysis confirmed that FLACS is associated with a more pronounced worsening of dry eye parameters in the early postoperative period compared with standard cataract surgery, although differences between techniques are no longer evident by three months [14]. A narrative review confirmed that cataract surgery can transiently destabilize the tear film and exacerbate DED, particularly in patients with meibomian gland dysfunction or diabetes, with some symptoms persisting for months [16].

This study aimed to compare ocular surface changes after FLACS and SCS, and to identify preoperative risk factors associated with postoperative dry eye symptoms using multivariable median quantile regression.

## 2. Methods

### 2.1. Study Design

In this prospective study, patients undergoing either SCS or FLACS between July and November 2024 were enrolled at the Ophthalmology Clinic of the Policlinico Umberto I University Hospital, Sapienza University of Rome. All research and measurements were conducted in accordance with the principles of the Declaration of Helsinki, and the study had been approved by the local ethics committee. All participants provided written informed consent before enrollment in the study.

Subjects were enrolled if the following inclusion criteria were present: (1) best corrected visual acuity (BCVA) reduced to ≥0.1 logMAR in the affected eye; (2) clinical evidence based on slit-lamp evaluation of cataracts graded as NC2 or higher, according to the Lens Opacities Classification System III (LOCS III); [17] (3) dilated pupil width of at least 6.0 mm; (4) the ability to provide written informed consent to participate in the study and undergo surgical procedures. When cataract affected both eyes, the right eye was included in the study.

Exclusion criteria were as follows: (1) history of refractive surgery or ocular surgery; (2) prior diagnosis of autoimmune diseases (e.g., ocular cicatricial pemphigoid, Sjogren’s Syndrome, Rheumatoid arthritis, Rosacea, Steven-Johnson Syndrome/Lyell’s Syndrome); (3) other concomitant ocular diseases (glaucoma, retinal detachment, maculopathy, uveitis); (4) pseudoexfoliation syndrome or zonular weakness; (5) corneal scars or other corneal diseases; (6) current therapy with antidepressants, hormonal therapy or other medical treatment that could affect the tear film; (7) history of radiotherapy, chemotherapy, or monoclonal antibody treatment; (8) OSDI score > 33 points; (9) Schirmer I test I < 5 mm/5 min; (10) evidence of moderate or severe meibomian gland dysfunction; (11) use of systemic or topical steroids or nonsteroidal anti-inflammatory within the previous 30 days.

### 2.2. Ocular Surface Evaluation

A comprehensive ophthalmic evaluation, including a detailed ocular surface assessment, was performed preoperatively and postoperatively, at one week, one month, and three months. Collected data included the Ocular Surface Disease Index (OSDI) questionnaire, Non-Invasive Break-Up Time (NI-BUT), Schirmer I test, BCVA, and slit-lamp evaluation with corneal fluorescein staining assessed using the Oxford score.

The OSDI questionnaire is a well-established and widely validated instrument designed to assess subjective symptoms in individuals with dry eye disease [18]. The questionnaire comprises twelve items that evaluate three domains: ocular symptoms, vision-related functional impairment, and environmental triggers. Each item is scored based on the frequency of symptom occurrence. The OSDI questionnaire was self-administered by patients after being adequately explained by trained staff, in line with the original validation study [18].

NI-BUT test was performed using the Sirius^TM^ corneal topography device (Costruzione Strumenti Oftalmici; CSO, S.r.l, Firenze, Italy) which records tear film stability by videokeratoscopy at 25 frames per second. The average noninvasive tear film break-up time measured in seconds (s) was collected.

The Schirmer I test was performed under natural lighting conditions. A Schirmer test strip measuring 5 mm by 35 mm (AIESI^®^, Tear Strips, Naples, Italy) was positioned at the junction of the middle and outer thirds of the lower conjunctival sac of the affected eye, without the use of anesthesia. After 5 min, the strip was removed, and the length of the wet portion of the strip was recorded.

Corneal fluorescein staining was used to assess ocular surface damage with sterile sodium fluorescein strips and observed by a slit lamp microscope under a wide cobalt blue illumination. The presence of fluorescein staining in each quadrant (superior temporal, inferior temporal, superior nasal, and inferior nasal) was observed and recorded according to the Oxford score [19].

### 2.3. Surgical Procedure

On the day of the surgery, preoperative instillation of tropicamide + phenylephrine (10 mg/mL + 5 mg/mL eye) drops were administered as one drop every fifteen minutes for a total of three times. Topical anesthesia was achieved with lidocaine hydrochloride (40 mg/mL) eye drops combined with preservative-free intracameral lidocaine (1%). The surgical field was disinfected with 10% povidone-iodine for the skin and eyelids, and 5% povidone-iodine on conjunctival fornices for three minutes.

All surgeries were performed by a single experienced surgeon (MM). In FLACS, the femtosecond laser (LenSx, Alcon Laboratories, Inc., Fort Worth, TX, USA) was used for corneal incisions (2.4 mm main, 1.2 mm side-ports), capsulorhexis (4.8–5.2 mm), and nucleus chopping (10 μJ) under suction. Phacoemulsification was then completed manually (Centurion Vision System, Alcon Laboratories, Inc., Fort Worth, TX, USA). Residual cortex was removed by bimanual irrigation/aspiration, and an intraocular lens was implanted in the capsular bag. Incisions were sealed with hydro-sutures, and intracameral cefuroxime (0.1 mL, 10 mg/mL) was given.

In SCS, a 2.4 mm clear corneal incision and two 1.2 mm side-ports were made. After viscoelastic injection, manual capsulorhexis, hydrodissection, and phacoemulsification were performed. The remaining steps matched the FLACS group.

After surgery, both groups received one drop of a combination of betamethasone 0.2% and chloramphenicol 0.5%, then ocular bandage was applied.

Postoperatively, both groups received one drop of betamethasone 0.2% + chloramphenicol 0.5%, followed by an eye bandage. All patients used a standardized regimen: chloramphenicol (5 mg/mL) four times daily for 7 days; dexamethasone sodium phosphate gel (1.5 mg/mL) four times daily for 7 days, then tapered weekly; ketorolac (5 mg/mL) twice daily from day 7 for 4 weeks.

Intraoperative parameters recorded included ultrasound percentage (US%), ultrasound time, and cumulative dissipated energy (CDE, product of mean US% and US time).

### 2.4. Statistical Analysis

Statistical analysis was performed using JASP (Version 0.19.3). When specific modules were not available, the integrated R console within JASP was used to run additional analyses [20].

The sample size was calculated a priori for an independent-samples t test comparing OSDI scores at one week between FLACS and SCS. As this was a pilot, hypothesis-generating study, we targeted only large early effects. Based on preliminary data (Cohen’s d ≈ 1.30), with α = 0.05 and power of 0.80, 10 eyes per group were sufficient. To account for possible 10% attrition, approximately 11 patients per group were planned. This design was not powered to detect small-to-moderate effects.

Continuous variables are reported as both medians with interquartile ranges (IQRs) and means with standard deviations (SD), to enhance interpretability, whereas categorical variables are presented as frequencies and percentages. Due to the sample size and the failure to meet the normality assumption for data distribution, non-parametric statistical tests were applied and a *p*-value of less than 0.05 was considered significant. Comparisons of parameter variations between the two study groups at each time point were conducted using the Mann–Whitney U test and within-group comparisons of medians across different time points were evaluated using the Friedman test, followed by Holm’s post hoc analysis. Correlations were explored using Spearman’s coefficient and displayed in heatmaps, where warm colors indicate negative and cool colors positive associations.

**Figure 1:**
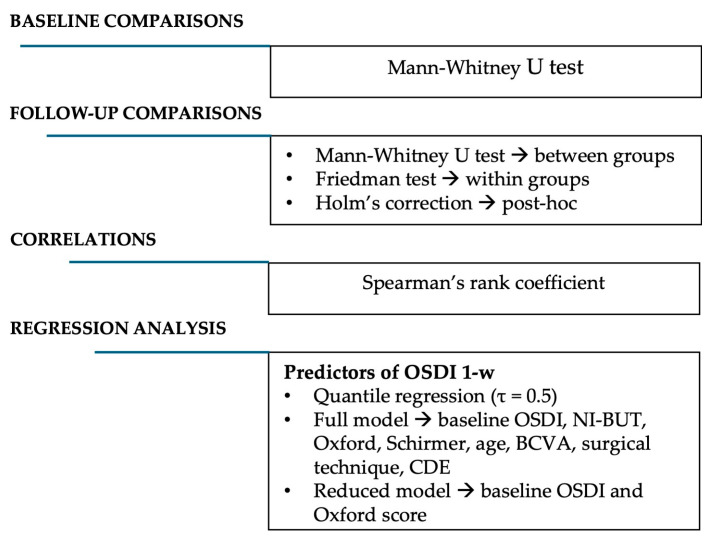
Summary of the statistical methods applied in the study.

Finally, to identify preoperative predictors of postoperative ocular surface discomfort, we performed a multivariable analysis using the OSDI score at one week as dependent variable. Given the small sample and non-normal distribution, multivariable quantile regression (median, τ = 0.5) was chosen over linear regression or mixed models. Independent variables considered a priori were baseline OSDI, NI-BUT, Oxford score, Schirmer I test, age, baseline BCVA, surgical technique (FLACS vs. SCS), and phacoemulsification parameters (US time, US%, CDE). Because of strong collinearity between CDE and US time (ρ = 0.89), CDE was retained as the synthetic indicator of ultrasonic energy, while US time and US% were excluded. Variance inflation factors (<4) confirmed acceptable collinearity. All predictors were entered simultaneously without stepwise selection (Figure 1).

## 3. Results

Twenty eyes of twenty patients were enrolled in the study, with ten undergoing FLACS and ten SCS. All surgeries were uneventful, and no intraoperative or major postoperative complications occurred during the three-month follow-up. Demographic and baseline characteristics for all patients are summarized in Table 1, while follow-up parameters are presented in Table 2.

Between-group differences were significant only for OSDI at 1 week (*p* = 0.023); no other differences were significant at baseline or subsequent time points (Table 1 and Table 2). Repeated-measures analysis confirmed a significant interaction between time and surgical technique for OSDI (F = 3.384, *p* = 0.047), indicating that the temporal pattern of symptoms differed between the FLACS and SCS groups. Holm-corrected post hoc tests showed higher OSDI in FLACS at one week, with no differences thereafter.

**Figure 2:**
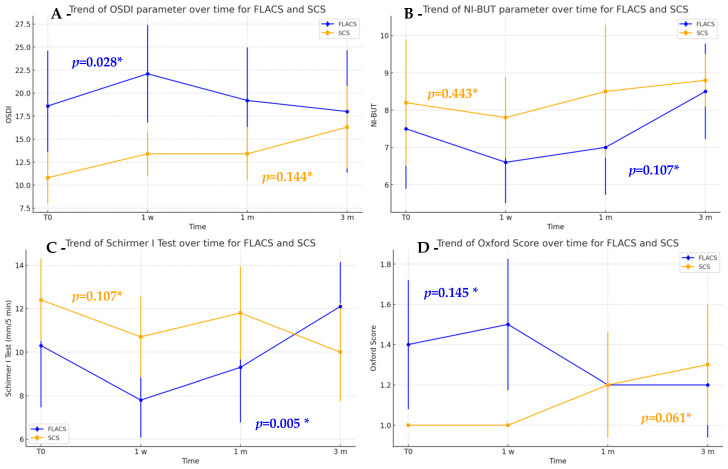
Descriptive plots showing the trends of OSDI (**A**), NI-BUT (**B**), Schirmer I Test (**C**) and Oxford score (**D**) in the FLACS (blue line) and SCS (orange line) groups. * Friedman Test.

Within-group analysis revealed that FLACS had a significant difference in OSDI scores over time (*p* = 0.028), with a peak at one week followed by a gradual decrease (Figure 2A). Post hoc tests confirmed the increase from baseline to one week (*p* = 0.036), followed by a decrease from one week to one month (*p* = 0.029). Similarly, the Schirmer I Test in the FLACS group showed a decrease at the one-week visit and subsequent improvement over time (*p* = 0.005). Post hoc analysis with Holm correction confirmed the initial reduction at one week and the significant improvement from one week to three months (*p* = 0.002) (Figure 2C). Although not statistically significant, the near-significant difference in Schirmer test pairwise values at 1 week (*p* = 0.092) suggests a potential trend that may be clinically relevant if confirmed in larger studies. No substantial differences were found for NI-BUT (Figure 2B) and Oxford score (Figure 2D).

**Figure 3:**
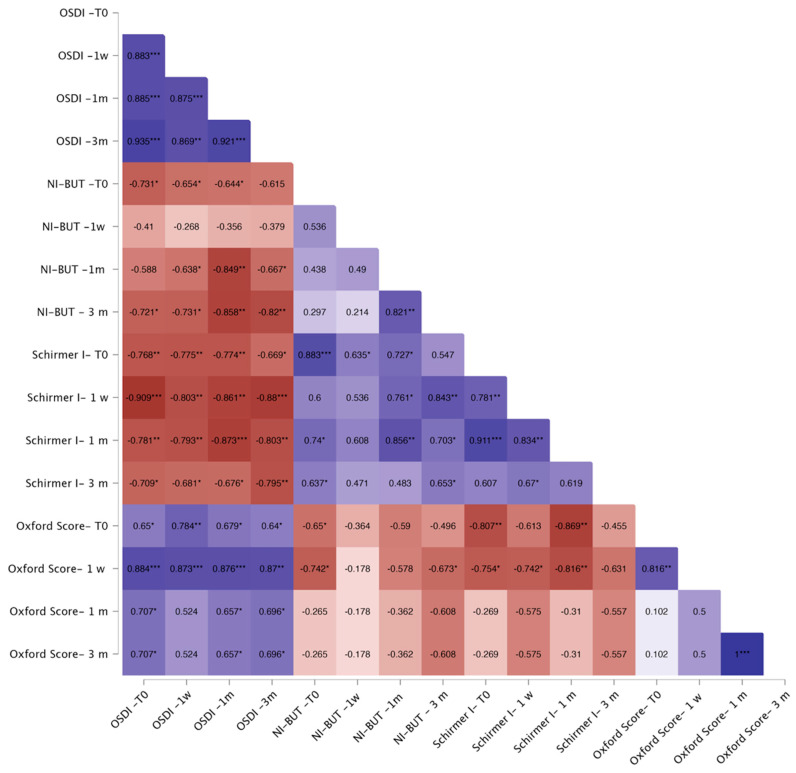
Correlation heatmap for the FLACS group. Warm colors (red) indicate stronger negative correlations, whereas cool colors (blue) indicate stronger positive correlations. Color intensity reflects the strength of correlation. The Spearman correlation coefficient is reported within each square of the heatmap. Significant correlations are indicated as follows: * *p* < 0.05; ** *p* < 0.01; *** *p* < 0.001.

**Figure 4:**
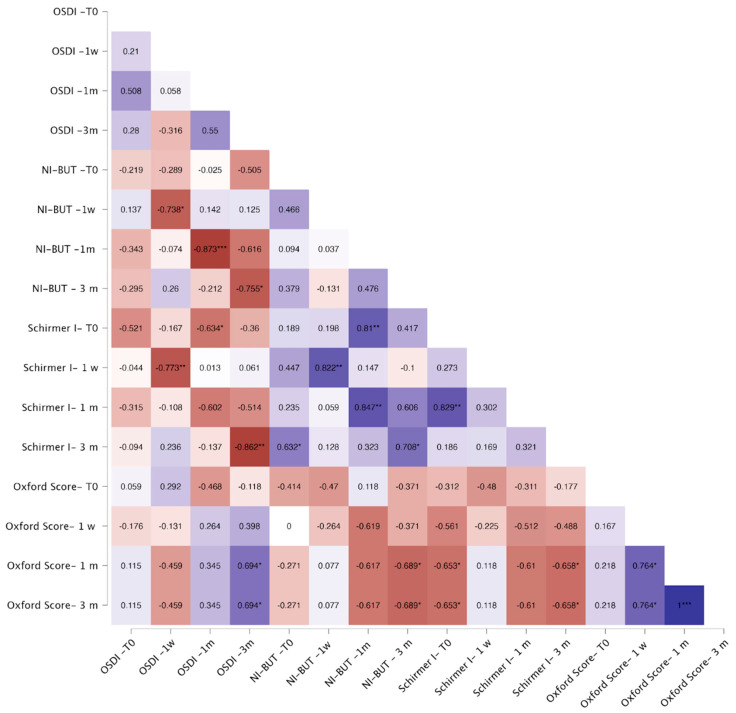
Correlation heatmap for the SCS group. Warm colors (red) indicate stronger negative correlations, whereas cool colors (blue) indicate stronger positive correlations. Color intensity reflects the strength of correlation. The Spearman correlation coefficient is reported within each square of the heatmap. Significant correlations are indicated as follows: * *p* < 0.05; ** *p* < 0.01; *** *p* < 0.001.

Correlation analyses showed that in the FLACS group, OSDI correlated negatively with baseline NI-BUT (*p* < 0.05) and Schirmer I (*p* < 0.01) at one week and one month, and with Schirmer I at three months (*p* < 0.05). Baseline Oxford score correlated positively with OSDI across all visits (*p* < 0.05, Figure 3). In the SCS group, OSDI showed no consistent correlations, except for a negative association with baseline Schirmer I at one month (*p* < 0.05) (Figure 4).

To assess preoperative predictors of postoperative ocular surface discomfort, we performed multivariable quantile regression (median, τ = 0.5) with OSDI at one week as the dependent variable. Independent variables specified a priori included baseline OSDI, NI-BUT, Oxford score, Schirmer I test, age, baseline BCVA, surgical technique (FLACS vs. SCS), and perioperative parameters (US time, US%, CDE); due to collinearity, only CDE was retained. In this full model, baseline OSDI (β = 0.61, 95% CI: 0.42–0.93, *p* < 0.001) was the strongest predictor, while baseline Oxford score showed an additional association, although with a large β estimate and wide confidence interval (β = 5.42, 95% CI: 0.12–10.73, *p* = 0.045). Surgical technique, perioperative factors, and demographic variables were not significant.

Given the limited sample size and the risk of overfitting, we then computed a reduced model focusing on two clinically relevant predictors: baseline OSDI and Oxford score. In this model, baseline OSDI remained significantly associated with postoperative OSDI at one week (β = 0.61, 95% CI: 0.40–0.82, *p* < 0.001), and baseline Oxford score also retained a significant association (β = 6.75, 95% CI: 3.18–10.32, *p* = 0.001).

## 4. Discussion

DED following cataract surgery can be a significant cause of visual discomfort and reduced quality of life. In this study, preoperative OSDI and Oxford scores were the key predictors of early postoperative symptoms, whereas surgical technique was not an independent predictor after adjustment. Our findings indicate that higher baseline OSDI and more severe Oxford scores were associated with a worsening of symptoms at one week.

In our cohort, OSDI differed significantly between FLACS and SCS at one week (*p* = 0.023), with distinct trends observed between them (Figure 2A). Specifically, FLACS group exhibited a transient worsening at one week (*p* = 0.028) that resolved by three months, consistent with previous findings [21,22]. In contrast, the SCS group showed no significant differences throughout the follow-up period, as also noted by Shao et al. [22].

As previously observed [14], FLACS was associated with a more pronounced transient increase in OSDI at one week, although surgical technique was not an independent predictor after adjustment and differences diminished by three months. This early effect is likely linked to the temporary use of the suction ring, which can stress limbal stem cells, conjunctival epithelium, and goblet cells. Consistently, Lin et al. emphasized that postoperative DED after FLACS is not solely related to mechanisms common to cataract surgery, but also to unique factors linked to the patient interface, such as suction ring–induced stress and laser-related tear film instability [15].

In the SCS group, OSDI scores showed a gradual postoperative increase without statistical significance, as the three-month confidence intervals overlapped with baseline values. The more pronounced difference between FLACS and SCS at one week indicates an early, transient effect, while the convergence of scores by three months suggests that long-term outcomes are primarily driven by preoperative ocular surface status rather than surgical technique.

Multivariable quantile regression showed that worsening of OSDI at one week was mainly associated with higher baseline OSDI and poorer Oxford scores. This indicates that both subjective symptoms and ocular surface staining at baseline are key determinants of early postoperative discomfort, with baseline OSDI emerging as the most consistent predictor. These easily assessable preoperative markers may therefore help identify patients at greater risk of postoperative DED symptoms. Neither surgical technique nor perioperative parameters, including CDE, were independent predictors, suggesting that the transient OSDI increase in FLACS patients likely reflects pre-existing ocular surface vulnerability rather than the surgical approach itself. In subgroup analyses, baseline OSDI predicted symptoms only in the FLACS group, while no predictors emerged in SCS; however, this pattern should be interpreted cautiously and confirmed in larger cohorts.

Based on existing literature, previously identified risk factors for DED symptoms following cataract surgery included preoperative subclinical DED, presence of pro-inflammatory cytokines on the ocular surface and intraoperative factors such as the use of iodine, intense light exposure, extended surgical time, use of lid speculum, corneal incisions [23,24] and CDE used during the procedure [23]. Our pilot study indicates that preoperative ocular surface status, particularly OSDI and Oxford scores, may help predict postoperative discomfort. These findings are preliminary given the small sample size and exclusion of severe DED, but they highlight the value of simple, low-cost tools for identifying at-risk patients. In practice, those with elevated scores may benefit from ocular surface optimization (e.g., lubrication or anti-inflammatory therapy) before surgery. Patient satisfaction has been shown to correlate closely with OSDI scores [24], and systematic screening can reveal a high prevalence of undiagnosed dry eye even without conventional risk factors [25]. Early recognition and targeted treatment, including preservative-free artificial tears, may therefore enhance both outcomes and satisfaction [26].

Our study has several limitations. The small sample size and short follow-up restricted our findings to the early postoperative phase, and the power calculation, based on a large assumed effect size, may have limited the detection of subtler differences. Moreover, preoperative assessments of tear osmolarity and qualitative measurements related to matrix metalloproteinase levels [27], inflammatory cytokines [28], corneal sensitivity via esthesiometry [29] and anterior segment tomographic or topographic imaging were not performed. Of note, several additional factors may have influenced our findings, including the use of fluids and light exposure during surgery, as well as postoperative eye drops treatment [30,31]. Unlike previous studies [11,12], we did not observe a clear difference in CDE between groups. This finding may reflect the limited sample size and should be interpreted with caution, as it does not rule out the potential ultrasound-sparing advantage of FLACS. In addition, all surgeries were performed by a single experienced surgeon, which ensured procedural consistency but may limit external validity. Finally, in the reduced model, baseline OSDI remained a strong predictor, while Oxford score also showed an association, though with wide confidence intervals call for confirmation in larger cohorts.

Nevertheless, the strengths of our study include its prospective design, the high adherence to a stringent protocol, and the application of severe inclusion criteria. However, the strict exclusion criteria, although necessary to reduce confounding, may limit the generalizability of our findings. In clinical practice, many patients present with coexisting ocular surface disease, which may influence postoperative outcomes. Further studies with larger cohort sizes, more heterogeneous populations and longer follow-up period are needed to confirm our findings and elucidate the mechanisms underlying the differences observed in this preliminary study.

## 5. Conclusions

In conclusion, this exploratory study highlights the importance of preoperative ocular surface status as key determinant of early dry eye disease symptoms following cataract surgery. Particularly, baseline OSDI and Oxford scores may be associated with early postoperative DED symptoms, while surgical technique itself was not an independent predictor. These preliminary findings suggest that careful preoperative screening and optimization of the ocular surface may be more relevant than the surgical approach or perioperative energy parameters in reducing the risk of postoperative DED symptoms and improving patient satisfaction.

## Figures and Tables

**Table 1:** Baseline characteristics comparison between FLACS and SCS groups. BCVA: best corrected visual acuity; OSDI: ocular surface disease index; NI-BUT: non-invasive break-up time; LOCS: Lens Opacities Classification System III; US: ultrasound; CDE: cumulative dissipated energy. * Mann–Whitney U test.

	FLACS Group (n = 10)		SCS Group (n = 10)		
	Mean ± SD	Median (IQR)	Mean ± SD	Median (IQR)	*p* Value *
Age (years)	75.0 ± 4.5	76.5 (3.0)	71 ± 7.7	72.0 (3.75)	0.125
BCVA–baseline (logMAR)	0.3 ± 0.18	0.22 (0.23)	0.48 ± 0.55	0.35 (0.16)	0.336
BCVA–3 m (logMAR)	0.01 ± 0.03	0 (0)	0.01 ± 0.03	0 (0)	1.000
OSDI-baseline	18.6 ± 9.7	19.5 (15.3)	10.80 ± 4.5	10.0 (3.3)	0.111
NI-BUT–baseline (s)	7.5 ± 2.6	6.5 (3.8)	8.2 ± 2.7	7.5(2.5)	0.564
Schirmer I test–baseline (mm/5′)	10.3 ± 4.6	10.0 (7.8)	12.4 ± 3.1	14.0 (5.0)	0.321
Oxford score-baseline	1.4 ± 0.5	1.0 (1.0)	1.0 ± 0.0	1.0 (0)	NA
LOCS III (NC)	3.5 ± 1.2	3.0 (1.8)	3.8 ± 0.9	4.0 (0.8)	0.530
US time (s)	68.7 ± 27.8	68.5 (31.8)	66.9 ± 32.2	70.0 (43.8)	0.940
US (%)	13.1 ± 2.9	12.4 (1.9)	16.3 ± 7.6	14.8 (5.0)	0.151
CDE	12.0 ± 13.3	8.6 (5.1)	9.5 ± 8.1	5.5 (10.4)	0.677
Torsional (%)	28.5 ± 10.6	30.0 (6.7)	43.4 ± 21.3	39.5 (22.4)	0.089

**Table 2:** Comparison of follow-up measures between FLACS and SCS groups. OSDI: ocular surface disease index; NI-BUT: non-invasive break-up time. * Mann–Whitney U test.

	FLACS Group (n = 10)		SCS Group (n = 10)		
	Mean ± SD	Median (IQR)	Mean ± SD	Median (IQR)	*p* Value *
OSDI–1 w	22.1 ± 8.6	21.0 (13.8)	13.4 ± 3.9	14.0 (3.8)	0.023
OSDI–1 m	19.2 ± 9.3	20.0 (14.3)	13.4 ± 4.7	12.0 (5.3)	0.172
OSDI–3 m	18.0 ± 10.7	16.5 (17.0)	16.3 ± 7.2	16.0 (5.5)	0.939
NI-BUT–1 w (s)	6.6 ± 1.8	6.5 (2.0)	7.8 ± 1.8	8.0 (2.8)	0.178
NI-BUT–1 m (s)	7.0 ± 2.1	7.0 (1.5)	8.5 ± 2.9	7.5 (3.5)	0.375
NI-BUT–3 m (s)	8.5 ± 2.1	9.5 (2.5)	8.8 ± 1.1	8.5 (2.0)	0.936
Schirmer I test–1 w (mm/5′)	7.8 ± 2.8	8.5 (2.0)	10.7 ± 3.0	9.5 (5.5)	0.092
Schirmer I test–1 m (mm/5′)	9.3 ± 4.1	10.0 (5.8)	11.8 ± 3.5	12.5 (6.0)	0.260
Schirmer I test–3 m (mm/5′)	12.1 ± 3.3	13.5 (5.8)	10.0 ± 3.6	8.5 (6.5)	0.183
Oxford score–1 w	1.5 ± 0.5	1.5 (1.0)	1.0 ± 0.0	1.0 (0)	0.185
Oxford score–1 m	1.2 ± 0.4	1.0 (0)	1.2 ± 0.4	1.0 (0)	0.651
Oxford score–3 m	1.2 ± 0.4	1.0 (0)	1.3 ± 0.5	1.0 (0.8)	0.651

## Data Availability

The data that supports the findings of this study are available from the corresponding author, F.G., upon reasonable request.

## References

[1] Kauh C.Y., Blachley T.S., Lichter P.R., Lee P.P., Stein J.D. (2016). Geographic Variation in the Rate and Timing of Cataract Surgery Among US Communities. JAMA Ophthalmol..

[2] Wang W., Yan W., Müller A., He M. (2017). A Global View on Output and Outcomes of Cataract Surgery With National Indices of Socioeconomic Development. Investig. Ophthalmol. Vis. Sci..

[3] Chan E., Mahroo O.A.R., Spalton D.J. (2010). Complications of cataract surgery. Clin. Exp. Optom..

[4] Stein J.D. (2012). Serious adverse events after cataract surgery. Curr. Opin. Ophthalmol..

[5] Li X.-M., Hu L., Hu J., Wang W. (2007). Investigation of Dry Eye Disease and Analysis of the Pathogenic Factors in Patients after Cataract Surgery. Cornea.

[6] Kasetsuwan N., Satitpitakul V., Changul T., Jariyakosol S. (2013). Incidence and Pattern of Dry Eye after Cataract Surgery. PLoS ONE.

[7] Miura M., Inomata T., Nakamura M., Sung J., Nagino K., Midorikawa-Inomata A., Zhu J., Fujimoto K., Okumura Y., Fujio K. (2022). Prevalence and Characteristics of Dry Eye Disease After Cataract Surgery: A Systematic Review and Meta-Analysis. Ophthalmol. Ther..

[8] Stapleton F., Alves M., Bunya V.Y., Jalbert I., Lekhanont K., Malet F., Na K.-S., Schaumberg D., Uchino M., Vehof J. (2017). TFOS DEWS II Epidemiology Report. Ocul. Surf..

[9] Naderi K., Gormley J., O’Brart D. (2020). Cataract surgery and dry eye disease: A review. Eur. J. Ophthalmol..

[10] Cho Y.K., Kim M.S. (2009). Dry Eye After Cataract Surgery and Associated Intraoperative Risk Factors. Korean J. Ophthalmol..

[11] Kolb C.M., Shajari M., Mathys L., Herrmann E., Petermann K., Mayer W.J., Priglinger S.M., Kohnen T.M. (2020). Comparison of femtosecond laser–assisted cataract surgery and conventional cataract surgery: A meta-analysis and systematic review. J. Cataract Refract. Surg..

[12] Menapace R., Schartmüller D., Röggla V., Reiter G.S., Leydolt C., Schwarzenbacher L. (2022). Ultrasound energy consumption and macular changes with manual and femtolaser-assisted high-fluidics cataract surgery: A prospective randomized comparison. Acta Ophthalmol..

[13] Yu Y., Hua H., Wu M., Yu Y., Yu W., Lai K., Yao K. (2015). Evaluation of dry eye after femtosecond laser–assisted cataract surgery. J. Cataract Refract. Surg..

[14] Chen W.-T., Chen Y.-Y., Hung M.-C. (2022). Dry Eye Following Femtosecond Laser-Assisted Cataract Surgery: A Meta-Analysis. J. Clin. Med..

[15] Lin B., Li D., Zhang L., Chen L., Gao Y. (2024). Postoperative dry eye following femtosecond laser-assisted cataract surgery: Insights and preventive strategies. Front. Med..

[16] Villani E., Catania A.G., Luccarelli S.V., Magnani F., Martone G., Zanzottera E., Lagali N. (2023). Dry eye and cataract surgery: Narrative review and recommendations for management. Eur. J. Ophthalmol..

[17] Chylack L.T., Wolfe J.K., Singer D.M., Leske M.C., Bullimore M.A., Bailey I.L., Friend J., McCarthy D., Wu S.Y. (1993). The Lens Opacities Classification System III. The Longitudinal Study of Cataract Study Group. Arch. Ophthalmol..

[18] Schiffman R.M. (2000). Reliability and Validity of the Ocular Surface Disease Index. Arch. Ophthalmol..

[19] Bron A.J., Evans V.E., Smith J.A. (2003). Grading of corneal and conjunctival staining in the context of other dry eye tests. Cornea.

[20] JASP Team (2025). JASP.

[21] Ju R.-H. (2019). Changes in ocular surface status and dry eye symptoms following femtosecond laser-assisted cataract surgery. Int. J. Ophthalmol..

[22] Shao D., Zhu X., Sun W., Cheng P., Chen W., Wang H. (2018). Effects of femtosecond laser-assisted cataract surgery on dry eye. Exp. Ther. Med..

[23] Jiang D., Xiao X., Fu T., Mashaghi A., Liu Q., Hong J. (2016). Transient Tear Film Dysfunction after Cataract Surgery in Diabetic Patients. PLoS ONE.

[24] Szakáts I., Sebestyén M., Tóth É., Purebl G. (2017). Dry Eye Symptoms, Patient-Reported Visual Functioning, and Health Anxiety Influencing Patient Satisfaction After Cataract Surgery. Curr. Eye Res..

[25] Giannaccare G., Borselli M., Rossi C., Carnovale Scalzo G., Pellegrini M., Vaccaro S., Scalia G., Lionetti G., Mancini A., Bianchi P. (2024). Noninvasive screening of ocular surface disease in otherwise healthy patients scheduled for cataract surgery. Eur. J. Ophthalmol..

[26] Gomes J.A.P., Santo R.M. (2019). The impact of dry eye disease treatment on patient satisfaction and quality of life: A review. Ocul. Surf..

[27] Schargus M., Ivanova S., Stute G., Dick H.B., Joachim S.C. (2020). Comparable effects on tear film parameters after femtosecond laser-assisted and conventional cataract surgery. Int. Ophthalmol..

[28] Massingale M.L., Li X., Vallabhajosyula M., Chen D., Wei Y., Asbell P.A. (2009). Analysis of Inflammatory Cytokines in the Tears of Dry Eye Patients. Cornea.

[29] Giovannetti F., Sacchetti M., Marenco M., Alisi L., Visioli G., Bruscolini A., Lambiase A. (2024). New disposable esthesiometer (KeraSense^®^) to improve diagnosis and management of neurotrophic keratitis. Ocul. Surf..

[30] Yanai R., Yamada N., Ueda K., Tajiri M., Matsumoto T., Kido K., Nakamura S., Saito F., Nishida T. (2006). Evaluation of povidone-iodine as a disinfectant solution for contact lenses: Antimicrobial activity and cytotoxicity for corneal epithelial cells. Contact Lens Anterior Eye.

[31] McGee H.T., Fraunfelder F. (2007). Toxicities of topical ophthalmic anesthetics. Expert. Opin. Drug Saf..

